# Application of machine learning methods in predicting schizophrenia and bipolar disorders: A systematic review

**DOI:** 10.1002/hsr2.962

**Published:** 2022-12-28

**Authors:** Mahdieh Montazeri, Mitra Montazeri, Kambiz Bahaadinbeigy, Mohadeseh Montazeri, Ali Afraz

**Affiliations:** ^1^ Department of Health Information Sciences, Faculty of Management and Medical Information Sciences Kerman University of Medical Sciences Kerman Iran; ^2^ Medical Informatics Research Center, Institute for Futures Studies in Health Kerman University of Medical Sciences Kerman Iran; ^3^ Department of Computer, Faculty of Fatimah Kerman Branch Technical and Vocational University Kerman Iran

**Keywords:** bipolar, machine learning, prediction, schizophrenia

## Abstract

**Background and Aim:**

Schizophrenia and bipolar disorder (BD) are critical and high‐risk inherited mental disorders with debilitating symptoms. Worldwide, 3% of the population suffers from these disorders. The mortality rate of these patients is higher compared to other people. Current procedures cannot effectively diagnose these disorders because it takes an average of 10 years from the onset of the first symptoms to the definitive diagnosis of the disease. Machine learning (ML) techniques are used to meet this need. This study aimed to summarize information on the use of ML techniques for predicting schizophrenia and BD to help early and timely diagnosis of the disease.

**Methods:**

A systematic literature search included articles published until January 19, 2020 in 3 databases. Two reviewers independently assessed original papers to determine eligibility for inclusion in this review. PRISMA guidelines were followed to conduct the study, and the Prediction Model Risk of Bias Assessment Tool (PROBAST) to assess included papers.

**Results:**

In this review, 1243 papers were retrieved through database searches, of which 15 papers were included based on full‐text assessment. ML techniques were used to predict schizophrenia and BDs. The main algorithms applied were support vector machine (SVM) (10 studies), random forests (RF) (5 studies), and gradient boosting (GB) (3 studies). Input and output characteristics were very diverse and have been kept to enable future research. RFs algorithms demonstrated significantly higher accuracy and sensitivity than SVM and GB. GB demonstrated significantly higher specificity than SVM and RF. We found no significant difference between RF and SVM in terms of specificity.

**Conclusion:**

ML can precisely predict results and assist in making clinical decisions‐concerning schizophrenia and BD. RF often performed better than other algorithms in supervised learning tasks. This study identified gaps in the literature and opportunities for future psychological ML research.

## 1 INTRODUCTION

Schizophrenia and bipolar disorder (BD) are critical and high‐risk inherited mental disorders that have debilitating symptoms.^1^ These disorders are among the severe psychiatric diseases that have many overlaps and similarities with each other and affect the patient's behavior in the family and society. According to the World Health Organization, these disorders are among the top 10 causes of disability worldwide.^2^ Schizophrenia and BD affect 3% of the world's population.^3^, ^4^ Patients with schizophrenia and BD have a higher mortality rate than the general population.^5^ One of the prominent causes of death in these patients is suicide. In Danish registers, the rate of suicide is reported as 7.8% in men and 4.9% in women with BD.^6^

There are Five key features that define schizophrenia and BD and other psychotic disorders. These include delusions, hallucinations, disorganized thinking (inferred from speech), grossly disorganized or abnormal motor behavior, and negative symptoms.^7^ As compared with other disorders, schizophrenia and BD specifically, the presence or absence of specific psychotic symptoms identified as first‐rank symptoms (auditory hallucinations; thought withdrawal, insertion, or interruption; thought broadcasting; somatic hallucinations; delusional perception; feelings or actions controlled by external agents) may be particularly helpful for making the diagnosis.^8^, ^9^, ^10^

Many patients with schizophrenia and BD experience a long clinical period.^7^ Symptoms of the disease begin between the ages of 16 and 30. These symptoms fall into three categories: positive (hallucinations, delusions, and mental disorders), negative (lack or absence of facial expressions, feelings of little pleasure, and decreased sense of speech), and cognitive (concentrating and maintaining difficulty).^8^, ^9^, ^10^ BD patients experience persistent changes in brain structures, such as enlargement of the third and lateral ventricles of the brain and a decrease in the volume of gray matter in the anterior and middle cerebral cortex, cortical and mesotemporal cortex, and decrease in the posterior abdominal callus volume.^11^ The economic costs associated with the disease vary from $94 million to $102 billion each year.^12^ Therefore, this disease imposes a heavy financial burden on the patients, their families, and society.^13^ Predicting the disease can go a long way in preventing it and controlling its costs. Since it takes an average of 10 years from the onset of the first symptoms to the definitive diagnosis of the disease,^14^ current approaches cannot effectively diagnose these diseases. Machine learning (ML) techniques are proposed as an effective tool to meet this need.

ML is a domain of artificial intelligence that allows computer algorithms to learn patterns by studying data directly without being explicitly programmed.^15^ Artificial intelligence using ML is entering the realm of medicine at an increasing pace and has been tested in various clinical applications ranging from diagnosis to outcome prediction.^16^ The utilization of ML techniques has many advantages, such as recognizing diseases, reducing physician decision‐making errors, reducing healthcare costs, and improving the performance of healthcare providers.^17^

Various models have contributed significantly to the health domain, from rule‐based systems to advanced ML models (deep learning). These models have been used in prediction, diagnosis, and treatment in healthcare, such as predicting survival in breast cancer,^18^ diagnosis and prognosis of COVID‐19,^19^, ^20^ level of lung cancer,^21^ etc. ML techniques are also used to diagnose, classify, and predict schizophrenia and BD.^22^, ^23^, ^24^, ^25^, ^26^ Several ML methods have been used to predict the negative symptoms of schizophrenia based on speech signals^22^, ^27^ and to predict the recurrence of schizophrenia.^24^ Also, many studies have been conducted on the extraction of various features of computed tomography scans and magnetic resonance imaging (MRI) images in the diagnosis and prevention of schizophrenia and BD.^28^, ^29^, ^30^

Several algorithms, such as random forests (RF), support vector machine (SVM), and gradient boosting (GB), have been frequently used in this area. The RF method is fast, adaptable, and reliable for mining high‐dimensional data. As the name suggests, an ensemble of many decision trees makes up a RF. The RF produces a classification for each tree, and the class voted on the most becomes the prediction.^31^ SVMs are linear models for classification and regression problems. Several practical problems can be solved through this technique, including linear and nonlinear problems. This algorithm generates a line or a hyperplane to classify the data into classes.^32^ A GB algorithm trains many models (typically decision trees) in sequential and additive order. The purpose of boosting is to transform weak classifiers into strong classifiers. Each new model in GB is designed to minimize prediction error as much as possible.^33^

This study aimed to conduct a systematic review of ML algorithms for predicting schizophrenia and BD to help early and timely diagnosis of the diseases to improve patients’ health.

## 2 MATERIALS AND METHODS

### 2.1 Information source and search

A systematic search was conducted in PubMed, Web of Science, and Scopus for relevant studies published before January 18, 2020. PRISMA guidelines were followed to conduct this study.^34^ Two groups of keywords related to: (A) ML and (B) schizophrenia and BD were used to search these databases. The keywords used to identify relevant papers are shown in Appendix 1.

### 2.2 Inclusion and exclusion criteria

All studies applying ML techniques for predicting schizophrenia and BDs were considered. We included original studies. The search was restricted to English‐language publications. Editorials, commentaries, letters, books, presentations, and conference papers were excluded. All types of review studies were also excluded to prevent duplication in data collection.

### 2.3 Study selection

The selection process was initiated by removing duplicated papers. Then, two authors (MM, MM) independently reviewed the titles and abstracts of all identified studies. The same authors independently reviewed the relevant papers (MM, MM). The disagreements were resolved through discussion and, if required, referred to a third researcher (KB). The reasons for the exclusion of each study were documented during the screening process of the papers. Rayyan QCRI systematic review, a free web and mobile application platform, was used for paper screening.^35^

We additionally evaluated reference lists of relevant papers for relevant publications.

### 2.4 Data extraction and synthesis

We developed an Excel data‐extraction form to extract specific details of each paper (Appendix 2). Two reviewers (MM, MM) Completed the form. This form consisted of study's location, data utilized, sample size, ML model, accuracy, sensitivity, specificity, area under the receiver operating characteristic curves (AUC), and precision (Table 1). A more detailed table of the PROBAST results is shown in Appendix 3.

**Table 1:** Characteristics of included studies

Disorder	Data utilized	What is predicted?	Sample size	Machine learning model	Accuracy (%)	Sensitivity (%)	Specificity (%)	AUC (%)	Precision (%)	Risk of bias	First author	Year
Schizophrenia	EEG	Having Schizophrenia	30	SVM	78.05	NR	NR	NR	NR	High	Taylor et al.^37^	2017
				GPC	80.49	NR	NR	87	NR			
Schizophrenia	sMRI, fMRI	Cognitive functions	324	Meta‐analytic cognitive priors	70.9 (For mental domains)	NR	NR	NR	NR	High	M. Karrer et al.^43^	2019
					70.8 (For experimental tasks)							
Schizophrenia	Symptomatic and MRI	Symptom severity	167	LR	NR	NR	NR	81	NR	Low	Talpalaru et al.^44^	2019
				SVM	NR	NR	NR	78	NR			
				RF	NR	NR	NR	75	NR			
Schizophrenia	MRI	Neurotic, and psychotic symptoms	34	SVM	94	76	100	NR	NR	Low	Zarogianni et al.^38^	2016
Schizophrenia	Biobank	Having Schizophrenia	1606:	RBF	NR	NR	NR	NR	NR	High	Bracher‐Smith et al.^41^	2019
			803 SCZ	SVM	NR	NR	NR	NR	NR			
			803 HC	RF	NR	NR	NR	NR	NR			
				GBM	NR	NR	NR	NR	NR			
				NN	NR	NR	NR	NR	NR			
				LR	NR	NR	NR	NR	NR			
Schizophrenia	Data	Psychotic relapse	864:	CART	63.8	71.0	44.8	NR	NR	Low	Fond et al.^24^	2019
			549 baseline									
			315 2 years									

Schizophrenia	Whole exome sequencing of genotypes and phenotypes	Having Schizophrenia	5090:	XGBoost	85.7	84.9	86.6	NR	86.9	Low	Trakadis et al.^42^	2018
			2545 SCZ	L1.Logistic	74.6	72.0	77.3	NR	76.0			
			2545 HC	SVM	70.7	70.8	70.6	NR	70.5			
				RF	81.7	82.0	81.3	NR	81.1			
Schizophrenia	MRI, fMRI	Having Schizophrenia	211:	Ridge	87	NR	NR	NR	NR	Unclear	Salvador et al.^29^	2019
			96 SCZ	Lasso	80	NR	NR	NR	NR			
			115 HC	RF	NR	NR	NR	NR	NR			
				GB	NR	NR	NR	NR	NR			
Schizophrenia	fMRI	Having Schizophrenia	86	SVM	94.12	1	89.47	94.73	NR	Low	Nimkar et al.^26^	2018
	MRI			C5.0 DT	91.18	1	84.21	91	NR			
				RF	91.18	1	84.21	92.1	NR			
				K‐NN	79.41	1	63.16	81.579	NR			
				LDA	79.41	80	78.95	79.47	NR			
				GP	92	1	864	92.4	NR			
				NB	85.29	86.67	84.21	85.43	NR			
Schizophrenia	fMRI	High‐symptomatic patients vs. non/low‐symptomatic	174:	EM	86.9	79.8	93.1	NR	91.9	Low	Kalmady et al.^47^	2019
			81 SCZ									
			93 HC									
Schizophrenia	sMRI	Having Schizophrenia	606:	SVM	69	69	68	74	NR	Low	de Pierrefeu et al.^40^	2018
			276 SCZ	Enet	71	73	68	76				
			330 HC	Enet‐TV	68	68	68	74				
Schizophrenia	fMRI	Having Schizophrenia	144:	NR	NR	NR	NR	NR	NR	High	Silva et al.^74^	2014
	sMRI		69 SCZ									
			75 HC									
Bipolar	MRI	Symptom severity	94:	SVR	NR	NR	NR	NR	NR	High	Sartori et al.^30^	2018
			35 BD									
			59 HC									
Schizophrenia or bipolar disorder	MRI	Predict risk of developing Schizophrenia or bipolar disorder in offsprings	185:	SVM	77	NR	NR	NR	NR	High	Hillegers et al.^39^	2018
			50 SCZ									
			82 BD									
			53 HC									
Schizophrenia Bipolar	MRI	Having Schizophrenia	606	SVM	69	69	68	74	NR	Low	Pierrefeu et al.^46^	2018
				ElasticNet	71	73	68	76	NR			
				GraphNet	70	69	71	75	NR			
				TV‐Enet	68	68	68	74	NR			

*Note*: “**+**” indicates high risk of bias/low concern regarding applicability; “**−**” indicates low risk of bias/high concern regarding applicability; “**?**” indicates unclear risk of bias/unclear concern regarding applicability; NR = not reported.

Abbreviations: AUC, area under the receiver operating characteristic curves; BD, bipolar disorder; CART, classification and regression tree; C5.0 DT, C5.0 Decision Tree; EEG, electroencephalography; EM, ensemble model; EN, elastic net; Enet, elasticnet‐total variation; Enet‐TV, elasticnet‐total variation; GB, gradient boosting; GBM, gradient boosting machines; GP, Gaussian process; GPC, Gaussian processes classifiers; HC, healthy controls; K‐NN, K‐nearest neighbor; LASS, least absolute shrinkage and selection operator; LDA, linear discriminant analysis; LR, multivariate logistic regression; L1.Logistic, Lasso regularized (L1) logistic regression; MRI, magnetic resonance imaging; NB, Naïve Bayes; NN, neural networks; ONR, one nonlinear regression; RBF, linear and radial basis function; RF, random forests; SCZ, schizophrenia; SVM, support vector machine; SVR, support vector regression; Xgboost, extreme gradient boosting.

### 2.5 Risk of bias (ROB) assessment

To assess the ROB, we used the Prediction Model Risk of Bias Assessment Tool (PROBAST).^36^ It is a tool for assessing the ROB and the applicability of diagnostic and prognostic prediction model studies. It includes 20 signaling questions across 4 domains (participants, predictors, outcome, and analysis). This explanation and elaboration document describes the rationale for including each domain and signaling question and guides researchers, reviewers, readers, and guideline developers to use them to assess the ROB and applicability concerns.

## 3 RESULTS

**Figure 1:**
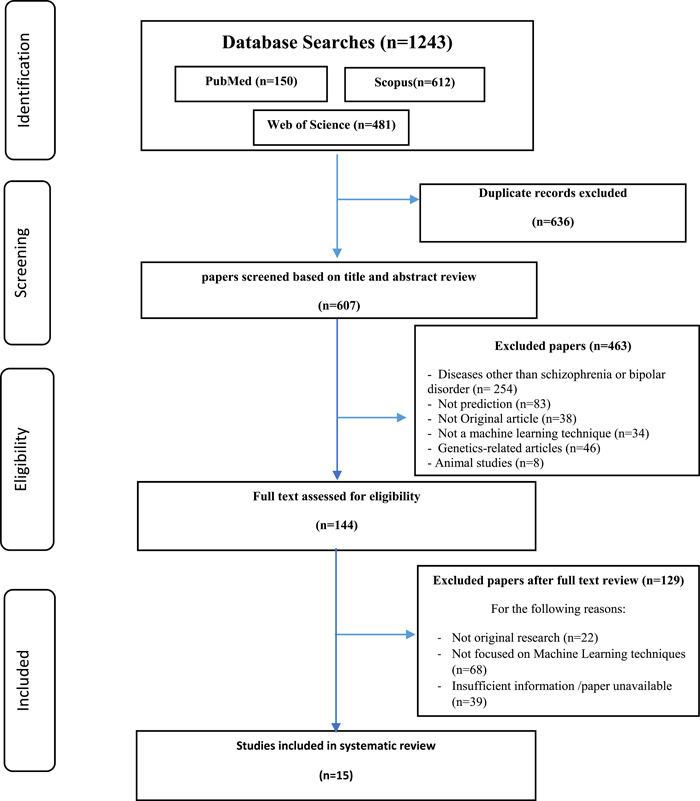
Study identification and selection process

We retrieved 1243 papers through database searches. After title and abstract screening 144 papers were identified for full‐text assessment. Full‐text assessment excluded 129 studies due to various reasons. Fifteen papers met the inclusion criteria (Figure 1). An analysis of the algorithms applied, the inputs they were trained on, the outputs they were trained to predict, and their relative performance statistics are presented. An average number of 185 patients were used in each study (mean = 681.4, *SD* = 1289.65). The median number of ML algorithms employed in each study was two (mean = 2.67, *SD* = 1.99). Most of the studies (*n* = 13) applied ML algorithms only to schizophrenia disorder, and one study applied ML algorithms to both schizophrenia and BDs. All the included studies applied ML algorithms to predict the symptoms of these disorders. Data utilizes in prediction of 10 studies was based on MRI. In eight studies, ML algorithms predicted schizophrenia disorder while in three studies these algorithms predicted symptoms severity.

### 3.1 Publications and algorithms applied in schizophrenia and BD over time

Over the past decade, many publications applying ML to schizophrenia and BD decision support have increased rapidly. The top two most frequently applied algorithms were SVM and RF. As shown in Table 1, most algorithms have been recently applied to schizophrenia and bipolar.

### 3.2 Summary of literature included, and algorithms applied

Most studies reported outcomes for more than one ML technique.^12^, ^38^, ^45^ The most frequently developed models used the variants of ML models, including SVM in 10 studies in references,^24^, ^30^, ^37^, ^38^, ^39^, ^40^, ^41^, ^42^, ^44^, ^46^ RF in 5 studies,^24^, ^29^, ^41^, ^42^, ^44^ GB in 3 studies^29^, ^41^, ^42^ with the  three different types of gradient boosting (GB) machines, Xgboost and GB, logistic regression in 3 studies^41^, ^42^, ^44^ and its extended L1.Logistic, decision tree in 2 studies^24^, ^26^ with two types of CART and C5.0 DT, and Gaussian process in 2 studies.^26^, ^37^ Several ML techniques each appeared in the one of the studies: Meta‐analytic cognitive prior,^43^ ElasticNet,^41^ GraphNet,^41^ TV‐Enet,^41^ linear and radial basis function,^42^ neural network,^42^ Ridge,^29^ Lasso,^29^ K‐NN,^26^ linear discriminant analysis,^26^ Naïve Bayes,^26^ and ensemble model.^47^

### 3.3 Predictive model performance evaluation statistics

The performances of the models were evaluated in varied ways. Accuracy and sensitivity (recall)‐specificity were the most frequently reported performance statistics (in 10 references^24^, ^26^, ^29^, ^37^, ^39^, ^40^, ^42^, ^43^, ^46^, ^47^ and 7 of studies,^24^, ^26^, ^38^, ^40^, ^42^, ^46^, ^47^ respectively). Five of the studies^26^, ^37^, ^40^, ^44^, ^46^ reported AUC, while Precision was reported by 2 studies.^42^, ^47^

### 3.4 Comparing algorithm performance

A comparison of the performance of the top three most frequently applied algorithms (SVM, RF, and GB) is shown in Table 2. Insufficient performance data were reported in this corpus to include other algorithms. We compared algorithms based on their accuracy, sensitivity, specificity, AUC, and Precision (Table 2). Multivariate logistic regression, Neural Network, and SVM differed significantly in their sensitivity performance. Based on this table, RF was significantly more accurate than SVM. The accuracy performance of RF and GB was not significantly different. The Specificity of the three algorithms was not significantly different. It showed that RF demonstrated significantly higher sensitivity than SVM.

**Table 2:** Statistical comparisons of reported performance metrics for support vector machine, random forests, and gradient boosting

	Performance metrics, mean (*SD*; *n*)				
	Accuracy (%)	Sensitivity (%)	Specificity (%)	AUC (%)	Precision (%)
Support vector machine (SVM)	80.48 (11.08, 6)	78.95 (14.34, 4)	82.02 (15.34, 4)	82.24 (11, 3)	70.5 (–, 1)
Random forests (RF)	86.44 (6.7, 2)	91 (12.73, 2)	82.75 (2.06, 2)	83.55 (12.09, 2)	81.1 (–, 1)
Gradient boosting (GB)	85.7 (–, 1)	84.9 (–, 1)	86.6 (–, 1)	–	86.9 (–, 1)

Abbreviation: AUC, area under the receiver operating characteristic curves.

### 3.5 Classification performance

The most commonly reported performance metrics for schizophrenia and BD were sensitivity (recall), specificity, and accuracy.^48^, ^49^ Multiple studies also presented positive predictive value (precision)^42^, ^47^ and error matrix outcomes as the AUC.^26^, ^37^, ^40^, ^44^, ^46^ However, there was no overall consistency as to which specific measures were reported.

### 3.6 Predictive model validation

In this study, 10 of the 13 studies provided details of a validation process for the applied models.^24^, ^26^, ^29^, ^30^, ^37^, ^38^, ^39^, ^40^, ^41^, ^42^, ^43^, ^46^, ^47^ Different forms of internal cross‐validation and holdout datasets were most commonly applied. *K‐fold* cross‐validation and leave‐one‐out cross‐validation frameworks were used to split data into training, validation, testing sets, or optimizing model parameters.^50^

### 3.7 Risk of bias

We critically reviewed studies for ROB using PROBAST.^36^ Our analysis revealed that except for six, all studies had some bias due to a low number of participants, lack of external validation, and failure to meet the study's goal (Table 1).

## 4 DISCUSSION

In this study, the applications of ML to support clinical decision‐making in schizophrenia and BD were reviewed. Results suggested that the use of ML in schizophrenia and BD is rapidly growing.^51^, ^52^ There is substantial room for further applications of ML technologies to schizophrenia and BD data. Many ML applications and modeling methods have been used based on the findings. A wide variety of successfully predicted outcomes could facilitate decision‐making. The number of ML publications on schizophrenia and BD has rapidly increased over the past few years.^53^, ^54^, ^55^

According to this study, most schizophrenia and BD ML publications were in the domain of MRI and fMRI data. Few ML studies have focused on EEG data. The applied modeling approaches were substantially heterogeneous. Variation in the studies was related to algorithms applied, input and output variables used, and methods for assessing predictive model performance.

In this paper, SVM and RF were the most commonly applied algorithms, compared to other studies, applying SVMS^56^, ^57^, ^58^, ^59^ and RF.^60^, ^61^ Consistent with a previous study,^62^ RF frequently outperformed most other algorithms on supervised learning tasks. Since both SVM and RF are discriminative, they can handle large amounts of data, and capture nonlinear relationships across input features.^44^ They were selected to predict disease outcomes, often demographics, clinical history, and investigation‐related features were used.^49^ AUC, sensitivity, accuracy, specificity, and precision performance metrics were the most commonly reported.^49^ The accuracy of RF was significantly higher than that of SVM, and compared to SVM, the precision of GB was significantly higher. To facilitate future modeling, the input and output variables were synthesized. Gaps in knowledge and opportunities concerning the development of additional clinical decision support systems to improve the care of schizophrenia and BD patients were highlighted to clinicians and data scientists. To our knowledge, this systematic review is the first attempt to characterize the deployment of ML methods in schizophrenia and BD. We identified two different topic clusters, and keywords for each of the schizophrenia and BD subdomains by applying ML technologies. The use of these topics and keywords in the systematic review helped to deeply understand the past foci of the relevant literature and determine the current state of the knowledge. This understanding helps to identify gaps to be filled out by future researches. ML provides a series of technologies, from linear regression to RF, that can effectively predict outcomes for improving clinical decision‐making in schizophrenia and BD. Based on the results, wherever large, labeled datasets were available, RF has been the top‐performing algorithm in the ML methods due to its higher accuracy than multivariate logistic regression.^62^

Since RF performed better than other models, it can be proposed for predicting schizophrenia and BD. It is fast to apply^63^ and suitable for feature selection (finding efficient risk factors) alone.^64^ RF does not need overtraining^65^ and can handle data without preprocessing, e.g., do not need rescaling, transforming, or modifying data.^65^ The following items are the notable performances of RF:

✓ Natural handling of “mixed” type data.^65^
✓ Handling of missing values.^66^
✓ Robustness to outliers in input space.^66^
✓ Insensitive to the monotone transformation of inputs.^67^
✓ Computational scalability.^68^
✓ Ability to deal with irrelevant inputs.^69^

Comparing RF with SVM and GB, it is true that they have about the same accuracy. However, RF is more interpretable due to these facts:

✓ Estimating feature importance during training for little additional computation.
✓ Plotting of sample proximities.^70^
✓ Visualizing output decision trees.^70^

RF is faster to train and can readily handle larger numbers of predictors. It employs fewer parameters^71^ and does not need cross‐validation (it generates an internal unbiased estimate of the generalization error (test error) as the forest building progresses).

## 5 LIMITATIONS AND FUTURE RESEARCH

It is more likely to publish positive and significant findings.^72^, ^73^ Many of the included studies evaluated the performance of multiple algorithms and reported the inferior performance of some. However, since we could not find studies reporting failed ML modeling activities, publication bias and selective outcome reporting may have influenced our results. Few studies have comprehensively reported predictive model performance statistics. Hence, we had relatively small sample sizes for our statistical analysis of algorithm performance. Larger sample sizes could have demonstrated additional significant performance differences between algorithms. More research is suggested to examine AUC, accuracy, sensitivity, and specificity to facilitate robust model performance evaluation and compare studies. Future studies can contribute to the convergence of ML with schizophrenia and BDs. Future studies can use similar predictors and outcomes on bigger databases containing the information of patients with schizophrenia and BDs. This may help predict the same or additional outcomes that can be used to deploy systems based on these models. The number of software to predict and support schizophrenia and BDs decision‐making is low. Further models have required these two disorders. Despite the richness of electronic health record datasets, they are not utilized sufficiently and are suitable for conducting ML studies. SVMs and transfer learning can utilize video data during real‐time patient examination, diagnosis and prognosis, or intraoperative anatomical identification. SVMs can predict the postoperative status of patients on an hourly basis to provide safer proactive management of patient status.

## 6 CONCLUSION

ML effectively supports making clinical decisions before, during, and after the onset of schizophrenia and BDs. Due to the evident heterogeneity of apply ML in psychology and the popularity of distinctive ML studies, there is a substantial potential for future similar studies. Because of the accurate prediction of various operative outcomes, RF seems more effective than other algorithms in schizophrenia and BDs. Deploying ML by designing sound clinical decision support systems can lower complications and increase the quality and safety of health services provided to schizophrenia and BD patients.

## AUTHOR CONTRIBUTIONS

**Mahdieh Montazeri**: project administration. **Mitra Montazeri**: formal analysis; writing—review & editing. **Kambiz Bahaadinbeigy**: conceptualization. **Mohadeseh Montazeri**: data curation; writing—original draft. **Ali Afraz**: conceptualization; writing—original draft.

## CONFLICTS OF INTEREST

The authors declare no conflicts of interest.

## TRANSPARENCY STATEMENT

The lead author Mitra Montazeri affirms that this manuscript is an honest, accurate, and transparent account of the study being reported; that no important aspects of the study have been omitted; and that any discrepancies from the study as planned (and, if relevant, registered) have been explained.

## Data Availability

The authors confirm that the data supporting the findings of this study are available within the article or its supplementary materials.

## APPENDIX 1 KEYWORDS USED TO IDENTIFY RELEVANT PAPERS

**PubMed**

**Web of Science**

**Scopus**

We included original studies. The search was restricted to English‐language publications. Editorials, commentaries, letters, books, presentations, and conference papers were excluded. All types of review studies were also excluded to prevent duplication in data collection. We had no filter on the date.

## APPENDIX 2 DATA EXTRACTION FORM

**Table:**

	A	B	C	D	E	F	G	H	I	J	K	L	M
1	**First author**	**Year**	**Data utilized**	**What is predicted?**	**Disorder**	**Sample size**	**Machine learning model**	**Accuracy (%)**	**Sensitivity (%)**	**Specificity (%)**	**AUC (%)**	**Precision (%)**	**Risk of bias**
2	Taylor et al.^34^	2017	EEG	having Schizophrenia	Schizophrenia	30	SVM	78.05	NR	NR	NR	NR	High

## APPENDIX 3 MORE DETAILED TABLE OF THE PROBAST RESULTS

**Table:**

Study	Risk of bias				
	Participants	Predictors	Outcome	Analysis	Overall
Taylor et al.^37^	Unclear	Low	Low	High	High
M. Karrer et al.^43^	Low	Low	Low	High	High
Talpalaru et al.^44^	Low	Low	Low	Low	Low
Zarogianni et al.^38^	Low	Low	Low	Low	Low
Hillegers et al.^39^	High	Unclear	Unclear	High	High
Pierrefeu et al.^46^	Low	Low	Low	Low	Low
Bracher‐Smith et al.^41^	High	Unclear	Unclear	High	High
Fond et al.^24^	Low	Low	Low	Low	Low
Trakadis et al.^42^	Low	Low	Low	Low	Low
Salvador et al.^29^	Unclear	Low	Low	Unclear	Unclear
Nimkar et al.^26^	Low	Low	Low	Low	Low
Kalmady et al.^47^	Low	Low	Low	Low	Low
Sartori et al.^30^	Unclear	Low	Low	High	High
de Pierrefeu et al.^40^	Low	Low	Low	Low	Low
Silva et al.^74^	Low	Low	Low	High	High

## References

[1] Cardno AG , Marshall EJ , Coid B , et al. Heritability estimates for psychotic disorders: the Maudsley twin psychosis series. Arch Gen Psychiatry. 1999;56(2):162‐168. 10025441 10.1001/archpsyc.56.2.162

[2] Heal A. A call for action by World Health Ministers. http://www.who.int/entity/mental_health/media/en/249.pdf (22 August 2015 dla).

[3] McGrath J , Saha S , Chant D , Welham J . Schizophrenia: a concise overview of incidence, prevalence, and mortality. Epidemiol Rev. 2008;30(1):67‐76. 18480098 10.1093/epirev/mxn001

[4] Merikangas KR , Akiskal HS , Angst J , et al. Lifetime and 12‐month prevalence of bipolar spectrum disorder in the national comorbidity survey replication. Arch Gen Psychiatry. 2007;64(5):543‐552. 17485606 10.1001/archpsyc.64.5.543 PMC1931566

[5] Crump C , Sundquist K , Winkleby MA , Sundquist J . Comorbidities and mortality in bipolar disorder: a Swedish national cohort study. JAMA Psychiatry. 2013;70(9):931‐939. 23863861 10.1001/jamapsychiatry.2013.1394

[6] Nordentoft M . Absolute risk of suicide after first hospital contact in mental disorder. Arch Gen Psychiatry. 2011;68(10):1058‐1064. 21969462 10.1001/archgenpsychiatry.2011.113

[7] Asken MJ , Grossman D , Christensen LW . American Psychiatric Association . *Diagnostic and statistical manual of mental disorders*. Arlington, VA: American Psychiatric Publishing, 2013. Archibald HC , and Tuddenham RD . Persistent stress reaction after combat: a 20‐year follow‐up. Arch General Psy Therapy . 2007(10):2317‐2325.

[8] Soares‐Weiser K , Maayan N , Bergman H , et al. First rank symptoms for schizophrenia (cochrane diagnostic test accuracy review): fig. 1. Schizophr Bull. 2015;41(4):792‐794. 25939661 10.1093/schbul/sbv061 PMC4466195

[9] Hegarty JD , Baldessarini RJ , Tohen M , Waternaux C , Oepen G . One hundred years of schizophrenia: a meta‐analysis of the outcome literature. Am J Psychiatry. 1994;151(10):1409‐1416. 8092334 10.1176/ajp.151.10.1409

[10] Kay SR , Fiszbein A , Opler LA . The positive and negative syndrome scale (PANSS) for schizophrenia. Schizophr Bull. 1987;13(2):261‐276. 3616518 10.1093/schbul/13.2.261

[11] Abé C , Ekman C‐J , Sellgren C , Petrovic P , Ingvar M , Landén M . Manic episodes are related to changes in frontal cortex: a longitudinal neuroimaging study of bipolar disorder 1. Brain. 2015;138(11):3440‐3448. 26373602 10.1093/brain/awv266

[12] Chong HY , Teoh SL , Wu DB‐C , Kotirum S , Chiou C‐F , Chaiyakunapruk N . Global economic burden of schizophrenia: a systematic review. Neuropsychiatr Dis Treat. 2016;12:357. 26937191 10.2147/NDT.S96649 PMC4762470

[13] Charlson FJ , Ferrari AJ , Santomauro DF , et al. Global epidemiology and burden of schizophrenia: findings from the global burden of disease study 2016. Schizophr Bull. 2018;44(6):1195‐1203. 29762765 10.1093/schbul/sby058 PMC6192504

[14] Lish JD , Dime‐Meenan S , Whybrow PC , Price RA , Hirschfeld RMA . The national depressive and manic‐depressive association (DMDA) survey of bipolar members. J Affect Disord. 1994;31(4):281‐294. 7989643 10.1016/0165-0327(94)90104-x

[15] Noble WS . What is a support vector machine? Nature Biotechnol. 2006;24(12):1565‐1567. 17160063 10.1038/nbt1206-1565

[16] Azimi P , Mohammadi HR , Benzel EC , Shahzadi S , Azhari S , Montazeri A . Artificial neural networks in neurosurgery. J Neurol Neurosurg Psychiatry. 2015;86(3):251‐256. 24987050 10.1136/jnnp-2014-307807

[17] Maroco J , Silva D , Rodrigues A , Guerreiro M , Santana I , de Mendonça A . Data mining methods in the prediction of Dementia: a real‐data comparison of the accuracy, sensitivity and specificity of linear discriminant analysis, logistic regression. neural networks, support vector machines, classification trees and random forests. BMC Res Notes. 2011;4:299. 21849043 10.1186/1756-0500-4-299 PMC3180705

[18] Montazeri M , Montazeri M , Montazeri M , Beigzadeh A . Machine learning models in breast cancer survival prediction. Technol Health Care. 2016;24(1):31‐42. 26409558 10.3233/THC-151071

[19] Montazeri M , ZahediNasab R , Farahani A , Mohseni H , Ghasemian F . Machine learning models for image‐based diagnosis and prognosis of COVID‐19: systematic review. JMIR Med Inform. 2021;9(4):e25181. 33735095 10.2196/25181 PMC8074953

[20] Montazeri M , Afraz A , Montazeri M , et al. Applications of artificial intelligence and machine learning in diagnosis and prognosis of COVID‐19 infection: a systematic review. Front Health Inform. 2021;10(1):93.

[21] Montazeri M , Afraz A , Mahboob Farimani R , Ghasemian F . Natural language processing systems for diagnosing and determining level of lung cancer: a systematic review. Front Health Inform. 2021;10(1):68.

[22] Chakraborty D , Xu S , Yang Z , et al. Prediction of negative symptoms of schizophrenia from objective linguistic, acoustic and non‐verbal conversational cues. 2018 International Conference on Cyberworlds (CW), 2018 Oct 3 (pp. 280‐283). IEEE.

[23] Winterburn JL , Voineskos AN , Devenyi GA , et al. Can we accurately classify schizophrenia patients from healthy controls using magnetic resonance imaging and machine learning? A multi‐method and multi‐dataset study. Schizophr Res. 2019;214:3‐10. 29274736 10.1016/j.schres.2017.11.038

[24] Fond G , Bulzacka E , Boucekine M , et al. Machine learning for predicting psychotic relapse at 2 years in schizophrenia in the national FACE‐SZ cohort. Prog Neuropsychopharmacol Biol Psychiatry. 2019;92:8‐18. 30552914 10.1016/j.pnpbp.2018.12.005

[25] Salvador R , Radua J , Canales‐Rodríguez EJ , et al. Evaluation of machine learning algorithms and structural features for optimal MRI‐based diagnostic prediction in psychosis. PLoS One. 2017;12(4):e0175683. 28426817 10.1371/journal.pone.0175683 PMC5398548

[26] Nimkar AV , Kubal DR , eds., Optimization of schizophrenia diagnosis prediction using machine learning techniques. 2018 4th International Conference on Computer and Information Sciences (ICCOINS). IEEE; 2018.

[27] Chakraborty D , Yang Z , Tahir Y , et al. Prediction of negative symptoms of schizophrenia from emotion related low‐level speech signals. 2018 IEEE International Conference on Acoustics, Speech and Signal Processing (ICASSP), 2018 Apr 15 (pp. 6024‐6028). IEEE.

[28] de Filippis R , Carbone EA , Gaetano R , et al. Machine learning techniques in a structural and functional MRI diagnostic approach in schizophrenia: a systematic review. Neuropsychiatr Dis Treat. 2019;15:1605. 31354276 10.2147/NDT.S202418 PMC6590624

[29] Salvador R , Canales‐Rodríguez E , Guerrero‐Pedraza A , et al. Multimodal integration of brain images for MRI based diagnosis in schizophrenia. Front Neurosci. 2019;13:1203. 31787874 10.3389/fnins.2019.01203 PMC6855131

[30] Sartori JM , Reckziegel R , Passos IC , et al. Volumetric brain magnetic resonance imaging predicts functioning in bipolar disorder: a machine learning approach. J Psychiatr Res. 2018;103:237‐43. 29894922 10.1016/j.jpsychires.2018.05.023

[31] Abdulkareem NM , Abdulazeez AM . Machine learning classification based on radom forest algorithm: a review. Int J Sci Bus. 2021;5(2):128‐142.

[32] Suthaharan S . Support vector machine. In Machine learning models and algorithms for big data classification. Springer; 2016:207‐235.

[33] Bahad P , Saxena P . Study of adaboost and gradient boosting algorithms for predictive analytics. In International Conference on Intelligent Computing and Smart Communication 2019. Springer; 2020:235‐244.

[34] Stewart LA , Clarke M , Rovers M , et al. Preferred reporting items for a systematic review and meta‐analysis of individual participant data: the PRISMA‐IPD statement. JAMA. 2015;313(16):1657‐1665. 25919529 10.1001/jama.2015.3656

[35] Ouzzani M , Hammady H , Fedorowicz Z , Elmagarmid A . Rayyan—a web and mobile app for systematic reviews. Syst Rev. 2016;5(1):210. 27919275 10.1186/s13643-016-0384-4 PMC5139140

[36] Wolff RF , Moons KGM , Riley RD , et al. PROBAST: a tool to assess the risk of bias and applicability of prediction model studies. Ann Intern Med. 2019;170(1):51‐58. 30596875 10.7326/M18-1376

[37] Taylor JA , Matthews N , Michie PT , Rosa MJ , Garrido MI . Auditory prediction errors as individual biomarkers of schizophrenia. NeuroImage: Clinical. 2017;15:264‐73. 28560151 10.1016/j.nicl.2017.04.027 PMC5435594

[38] Zarogianni E , Storkey AJ , Johnstone EC , Owens DGC , Lawrie SM . Improved individualized prediction of schizophrenia in subjects at familial high risk, based on neuroanatomical data, schizotypal and neurocognitive features. Schizophr Res. 2017;181:6‐12. 27613509 10.1016/j.schres.2016.08.027

[39] Hillegers M , Schnack H , Kahn R , Binnewies J , eds. Individual prediction of risk in adolescent offspring of parents with schizophrenia or bipolar disorder: a machine‐learning neuroimaging study with a cross‐stage validation. 65th Annual Meeting; 2018: AACAP.

[40] de Pierrefeu A , Löfstedt T , Laidi C et al., eds. Interpretable and stable prediction of schizophrenia on a large multisite dataset using machine learning with structured sparsity. 2018 International Workshop on Pattern Recognition in Neuroimaging (PRNI); 2018: IEEE.

[41] Bracher‐Smith M , Menzies G , Kendall K , et al. F29investigating supervised machine learning methods for prediction of schizophrenia in uk biobank. Eur Neuropsychopharmacol. 2019;29:S1125.

[42] Trakadis YJ , Sardaar S , Chen A , Fulginiti V , Krishnan A . Machine learning in schizophrenia genomics, a case‐control study using 5,090 exomes. Am J Med Genet, Part B. 2019;180(2):103‐112. 29704323 10.1002/ajmg.b.32638

[43] Karrer TM , Bassett DS , Derntl B , et al. Brain‐based ranking of cognitive domains to predict schizophrenia. Hum Brain Mapp. 2019;40(15):4487‐507. 31313451 10.1002/hbm.24716 PMC6865423

[44] Talpalaru A , Bhagwat N , Devenyi GA , Lepage M , Chakravarty MM . Identifying schizophrenia subgroups using clustering and supervised learning. Schizophr Res. 2019;214:51‐59. 31455518 10.1016/j.schres.2019.05.044

[45] Darminto MR , Chu HJ . Mapping landslide release area using random forest model. IOP Conf Ser: Earth Environ Sci. 2019;389(1):012038.

[46] de Pierrefeu A , Löfstedt T , Laidi C , et al. Identifying a neuroanatomical signature of schizophrenia, reproducible across sites and stages, using machine learning with structured sparsity. Acta Psychiatr Scand. 2018;138(6):571‐580. 30242828 10.1111/acps.12964

[47] Kalmady SV , Greiner R , Agrawal R , et al. Towards artificial intelligence in mental health by improving schizophrenia prediction with multiple brain parcellation ensemble‐learning. NPJ Schizophr. 2019;5(1):2. 30659193 10.1038/s41537-018-0070-8 PMC6386753

[48] Tharwat A . Classification assessment methods. Appl Comput Inform. 2020;17:1.

[49] Buchlak QD , Esmaili N , Leveque J‐C , et al. Machine learning applications to clinical decision support in neurosurgery: an artificial intelligence augmented systematic review. Neurosurg Rev. 2020;43(5):1235‐53. 31422572 10.1007/s10143-019-01163-8

[50] Brownlee J . A gentle introduction to k‐fold cross‐validation. Machine Learning Mastery. 2018;17:2019.

[51] Karthik S , Sudha M . Predicting bipolar disorder and schizophrenia based on non‐overlapping genetic phenotypes using deep neural network. Evol Intell. 2021;14(2):619‐634.

[52] CIaude LA , Houenou J , Duchesnay E , Favre P . Will machine learning applied to neuroimaging in bipolar disorder help the clinician? A critical review and methodological suggestions. Bipolar Disord. 2020;22(4):334‐55. 32108409 10.1111/bdi.12895

[53] Bracher‐Smith M , Crawford K , Escott‐Price V . Machine learning for genetic prediction of psychiatric disorders: a systematic review. Mol Psychiatry. 2021;26(1):70‐79. 32591634 10.1038/s41380-020-0825-2 PMC7610853

[54] Achalia R , Sinha A , Jacob A , et al. A proof of concept machine learning analysis using multimodal neuroimaging and neurocognitive measures as predictive biomarker in bipolar disorder. Asian J Psychiatr. 2020;50:101984. 32143176 10.1016/j.ajp.2020.101984

[55] Abohamza E , Weickert T , Ali M , Moustafa AA . Reward and punishment learning in schizophrenia and bipolar disorder. Behav Brain Res. 2020;381:112298. 31622639 10.1016/j.bbr.2019.112298

[56] Alonso SG , De la Torre Díez I , Hamrioui S , et al. Data mining algorithms and techniques in mental health: a systematic review. J Med Syst. 2018;42(9):161. 30030644 10.1007/s10916-018-1018-2

[57] Caballé NC , Castillo‐Sequera JL , Gómez‐Pulido JA , Gómez‐Pulido JM , Polo‐Luque ML . Machine learning applied to diagnosis of human diseases: a systematic review. Appl Sci. 2020;10(15):5135.

[58] Islam M , Hasan M , Wang X , Germack H , Noor‐E‐Alam M . A systematic review on healthcare analytics: application and theoretical perspective of data mining. Healthcare. 2018;6(2):54. 29882866 10.3390/healthcare6020054 PMC6023432

[59] Gupta A , Katarya R . Social media based surveillance systems for healthcare using machine learning: a systematic review. J Biomed Inf. 2020;108:103500. 10.1016/j.jbi.2020.103500 PMC7331523 32622833

[60] Wang W , Kiik M , Peek N , et al. A systematic review of machine learning models for predicting outcomes of stroke with structured data. PLoS One. 2020;15(6):e0234722. 32530947 10.1371/journal.pone.0234722 PMC7292406

[61] Liang H , Yang L , Tao L , et al. Data mining‐based model and risk prediction of colorectal cancer by using secondary health data: a systematic review. Chin J Cancer Res. 2020;32(2):242‐251. 32410801 10.21147/j.issn.1000-9604.2020.02.11 PMC7219096

[62] Sarica A , Cerasa A , Quattrone A . Random forest algorithm for the classification of neuroimaging data in Alzheimer's disease: a systematic review. Front Aging Neurosci. 2017;9:329. 29056906 10.3389/fnagi.2017.00329 PMC5635046

[63] Ziegler A , König IR . Mining data with random forests: current options for real‐world applications. Wiley Interdisciplinary Reviews: Data Mining and Knowledge Discovery. 2014;4:55‐63.

[64] Fennell PG , Zuo Z , Lerman K . Predicting and explaining behavioral data with structured feature space decomposition. EPJ Data Science. 2019;8(1):23.

[65] Akhil J , Deekshatulu B , Chandra P . Intelligent heart disease prediction system using random forest and evolutionary approach. JNIC. 2016;4:175‐184.

[66] Carvajal G , Maucec M , Cullick S . Chapter four ‐ components of artificial intelligence and data analytics. In: Carvajal G , Maucec M , Cullick S , eds. Intelligent Digital Oil and Gas Fields. Gulf Professional Publishing; 2018:101‐148.

[67] Galili T , Meilijson I . Splitting matters: how monotone transformation of predictor variables may improve the predictions of decision tree models. arXiv preprint arXiv:161104561. 2016.

[68] te Beest DE , Mes SW , Wilting SM , Brakenhoff RH , van de Wiel MA . Improved high‐dimensional prediction with random forests by the use of co‐data. BMC Bioinformatics. 2017;18(1):584. 29281963 10.1186/s12859-017-1993-1 PMC5745983

[69] Schöning V . *Machine learning for the prediction of drug‐induced toxicity*. University of Basel; 2019.

[70] Ruiz‐Samblás C , Cadenas JM , Pelta DA , Cuadros‐Rodríguez L . Application of data mining methods for classification and prediction of olive oil blends with other vegetable oils. Anal Bioanal Chem. 2014;406(11):2591‐2601. 24577575 10.1007/s00216-014-7677-z

[71] Wang L , Zhou X , Zhu X , Dong Z , Guo W . Estimation of biomass in wheat using random forest regression algorithm and remote sensing data. Crop J. 2016;4(3):212‐219.

[72] Chan A‐W , Hróbjartsson A , Haahr MT , Gøtzsche PC , Altman DG . Empirical evidence for selective reporting of outcomes in randomized trials: comparison of protocols to published articles. JAMA. 2004;291(20):2457‐2465. 15161896 10.1001/jama.291.20.2457

[73] Song F , Parekh‐Bhurke S , Hooper L , et al. Extent of publication bias in different categories of research cohorts: a meta‐analysis of empirical studies. BMC Med Res Methodol. 2009;9(1):79. 19941636 10.1186/1471-2288-9-79 PMC2789098

[74] Silva RF , Castro E , Gupta CN , et al., eds. The tenth annual MLSP competition: schizophrenia classification challenge. 2014 IEEE International Workshop on Machine Learning for Signal Processing (MLSP); IEEE; 2014.

